# One year monitoring of SARS-CoV-2 prevalence in a German cohort of patients with cystic fibrosis

**DOI:** 10.1186/s12890-022-01900-8

**Published:** 2022-03-24

**Authors:** Anke Jaudszus, Mariya Pavlova, Marius Rasche, Michael Baier, Anne Moeser, Michael Lorenz

**Affiliations:** 1 Cystic Fibrosis Centre, Department of Paediatrics, Jena University Hospital, Friedrich-Schiller-University, Am Klinikum 1, 07747 Jena, Germany; 2 Institute for Infectious Diseases and Infection Control, Jena University Hospital, Friedrich-Schiller-University, Jena, Germany; 3 Institute of Medical Microbiology, Jena University Hospital, Friedrich-Schiller-University, Jena, Germany

## Abstract

**Background:**

In Germany, the first case of coronavirus disease 2019 (COVID-19) was registered on 28 January 2020. By February 2021, the third wave of the pandemic began. So far, only few data are available on the SARS-CoV-2 prevalence and the clinical impact of an infection in patients with cystic fibrosis (CF).

**Methods:**

From February 2020 until March 2021, we screened 156 CF patients for anti-SARS-CoV-2 IgG antibodies (serology) and the presence of SARS-CoV-2 in deep throat saliva or nasopharyngeal swabs (molecular testing). From patients with confirmed SARS-CoV-2 infection, we recorded symptoms and collected clinical data.

**Results:**

In total, 13 patients (8.3%) were tested positive for SARS-CoV-2 infection, most of them during the second and the beginning third wave of the pandemic. Ten positive tested patients described symptoms linked to COVID-19. The most common symptom was cough followed by fatigue and headache. SARS-CoV-2 infection did not impair lung function. No positive tested patient needed to be hospitalized.

**Conclusions:**

SARS-CoV-2 infections in patients with CF are not as rare as initially anticipated, as frequent testing revealed. However, infected patients did not experience more severe clinical courses or worse clinical outcome. Our observation is in line with published reports indicating that individuals with CF are not at higher risk for severe COVID-19.

## Introduction

Severe acute respiratory syndrome coronavirus type 2 (SARS-CoV-2) is a novel beta coronavirus that was identified in early 2020 as causative of coronavirus disease 2019 (COVID-19). First appeared in December 2019 in Wuhan, China [1], SARS-CoV-2 has rapidly spread throughout the world. Globally, as of 3 May 2021, there have been 157.034.286 confirmed cases, including 3.389.504 deaths, reported to the world health organization [2]. Three quarters of a year later, as of 18 January 2022, the confirmed cases of COVID-19 have doubled with now 328.532.929, including 5.542.359 deaths [2]. In Germany, the first case was registered on 28 January 2020 [3]. The peak of the first wave was reached in March 2020. In mid-October, the second wave of the pandemic hit Germany with a dramatic increase in the number of infections. In February 2021, the third wave began with even faster progression.

Cystic fibrosis (CF) is the most common inherited life-shortening disease in Caucasians. This multisystemic disorder, affecting airways and gastrointestinal tract, is caused by mutations of the gene encoding for the cystic fibrosis transmembrane regulator (CFTR), the main transmembrane transporter for chloride and bicarbonate in the apical membrane of secretory and absorptive epithelial cells. In the airways, defects in CFTR lead to impaired mucociliary clearance, pathogen infection, and inflammation. Progressive lung destruction triggered by chronic pulmonary infection with opportunistic pathogens is the leading cause of premature death in CF [4]. Preexisting medical conditions such as chronic obstructive pulmonary disease (COPD) have been identified as risk factors for worse clinical outcomes of SARS-CoV-2 infection [5]. Whether patients with CF are a potentially highly vulnerable group to COVID-19 is yet to be clarified. In this prospective study, we present data on prevalence and clinical impact of SARS-CoV-2 infection in 156 patients with CF monitored over one year of the pandemic.

## Methods

### Study population

By February 2020, we started to monitor our patients during their regular visits which usually take place every 3–6 months. Of the 175 CF patients attended at the CF Center of the Jena University Hospital, 156 patients participated in the monitoring (Table 1). Of all tested patients, 132 (84.6%) were tested at least twice during the monitoring period. Between February 2020 and March 2021, we analyzed a total of 276 blood samples by serological tests and a total of 191 deep throat saliva or nasopharyngeal swab specimens by real-time polymerase chain reaction (RT-PCR). Specific antibodies were analyzed from regular routine blood withdrawals. PCR tests were performed when patients reported an acquired infection or respiratory exacerbation. During the study period, 6 patients have been vaccinated.

**Table 1:** Patient characteristics of the study cohort

Variable	Frequency	
	*n*	%
Gender		
Male	71	45.5
Female	85	54.5
Age (y)		
< 18	70	44.9
≥ 18	86	55.1
*CFTR* genotype		
*F508del/F508del*	63	40.4
*F508del/G551D*	15	9.6
*F508del/other*	61	39.1
*Other/other*	17	10.9
CF-related diabetes^a^	58	37.2

	Mean ± SD	Median
FEV1pp (n = 136 ^b^)	93.2 ± 27.0	98.0
BMI^c^	20.0 ± 3.9	20.1

Definition of abbreviations: y, years; CFTR, cystic fibrosis transmembrane conductance regulator; FEV1pp, forced expiratory volume in one second, percent predicted; BMI, body mass index; SD, standard deviation

^a^Glycosylated hemoglobin ≥ 6.5%, fasting plasma glucose ≥ 7 mmol/L, and/or 2-h oral glucose tolerance test (oGTT) plasma glucose ≥ 11 mmol/L.; ^b^n is specified when data are missing (no measurements in children < 6 y); ^c^weight classification of children according to S2 AGA 10/2015 guideline [6] using gender-specific age percentiles

### SARS-CoV-2 antibody testing

Antibodies against SARS-CoV-2 were quantitatively detected by two different commercially available chemiluminescence immunoassays: LIAISON SARS-CoV-2 S1/S2 IgG (DiaSorin, Saluggia, Italy), designed for antibodies against the S1- and S2 domain and Elecsys Anti-SARS-CoV-2 (Roche, Mannheim, Germany) for antibodies against the nucleocapsid protein (N). Both assays were carried out according to the manufacturers’ instructions. As provided by the manufacturers, sensitivity (> 97.5% in samples taken 15 days after positive RT-PCR result) and specificity (≥ 98%) are high for both tests. In case of two corresponding negative test results by both immunoassays, the patient was regarded as SARS-CoV-2 seronegative. Seropositivity after natural infection was assumed when both immunoassay results were positive.

### SARS-CoV-2 RT-PCR testing

Detection of SARS-CoV-2 in deep throat saliva or nasopharyngeal swab was performed by RT-PCR amplification of SARS-CoV-2 E-gene (RNA-extraction: QIASymphony instrument, QIAsymphony DSP Virus/Pathogen MiniKit (Qiagen, Hilden, Germany), amplification/detection: LightCycler 480 II instrument (F. Hoffmann-La Roche AG, Basel, Switzerland), LightMix Modular Sarbecovirus E-gene kit (TIB MOLBIOL, Berlin, Germany) and N/Nsp-gene (NeuMoDx SARS-CoV-2 Assay, NeuMoDx Molecular, Ann Arbor, USA) according to the manufacturers instructions.

## Results

### SARS-CoV-2 positive findings

**Fig. 1:**
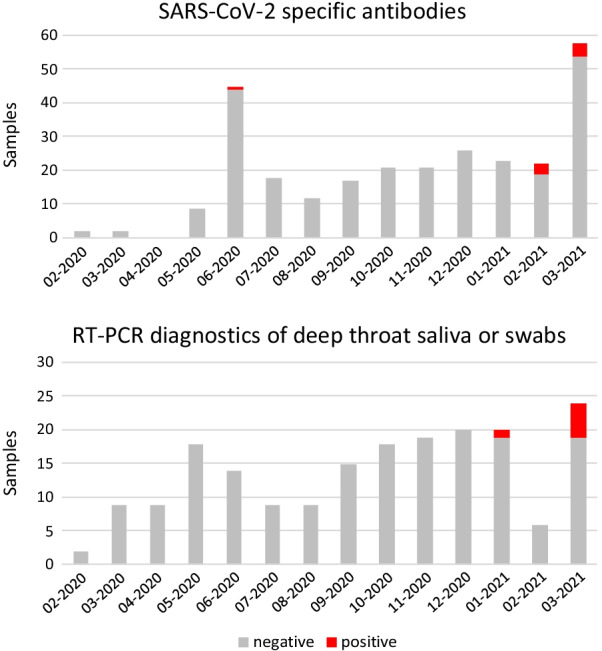
Serologies and molecular testing against SARS-CoV-2 during the monitoring period. Patients were tested during their routine visits

In total, 8 of 156 tested patients (5.1%) showed positive serology against SARS-CoV-2. After adjusting for test performance [8], the SARS-CoV-2 seroprevalence in this study was 4.2% (95% confidence interval 1.7–8.9%). Six patients had a SARS-CoV-2 positive RT-PCR testing, in one of them seroconversion was confirmed during the next routine visit. Overall, 13 patients (8.3%) were directly or indirectly tested positive for SARS-CoV-2. The majority of the positive findings occurred during the second wave of the pandemic (January–March 2021; Fig. 1). All PCR positive patients were confirmed seropositive in the further course.

### Symptoms

Sixty-five patients (41.7%) reported on at least one respiratory exacerbation during the monitoring period. As CF-associated respiratory symptoms might have been compatible with COVID-19, it was of particular interest to determine whether the symptoms were linked to a SARS-CoV-2 infection. We were able to screen 35 of these symptomatic patients at the time of exacerbation or within the following 2 weeks. In the tested patients, no respiratory exacerbation coincided with SARS-CoV-2 positive finding.

**Fig. 2:**
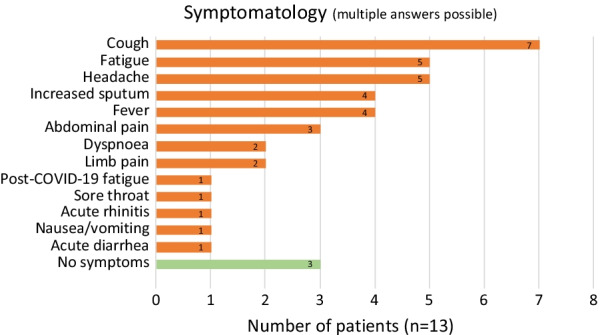
Reported symptoms during SARS-CoV-2 infection (n = 13, multiple answers possible)

The cumulative incidence of SARS-CoV-2 infection was 8.3%. In 6 children (age range 2–9 years) and 7 adults, a SARS-CoV-2 infection was confirmed. Among them, 10 patients reported on symptoms attributed to COVID-19, whereas 3 patients (2 pediatric and one adult) went through the infection without symptoms. In both pediatric and adult patients, the most common symptom was cough (53.8%) followed by fatigue and headache (38.5% each; Fig. 2). Of the 6 infected pediatric patients, 3 reported on cough and fatigue, and 2 of them additionally on fever and headache. None of the symptomatic children had limb pain, sore throat, nausea, or diarrhea. None of the SARS-CoV-2 infected patients needed to be hospitalized. In general, among the infected patients no one was post-transplant.

### Clinical impact

**Fig. 3:**
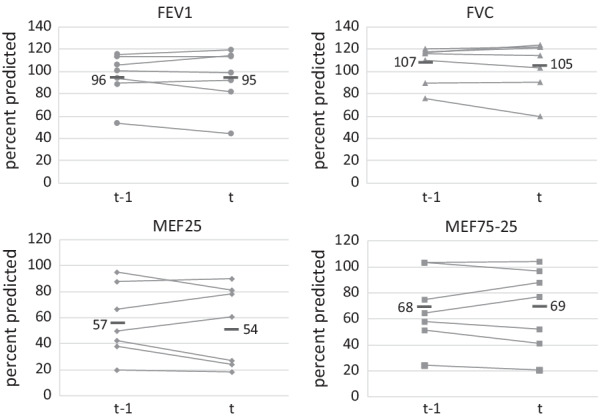
No changes in lung function parameters prior to (t-1) and with/after SARS-CoV-2 infection (t). Measurement in patients > 6 years. *FEV1* forced expiratory volume in one second, *FVC* forced vital capacity, *MEF25* maximum expiratory flow at 25% of FVC, *MEF75-25* maximum midexpiratory flow between 75 and 25% of FVC

With respect to lung function parameters, no impact of SARS-CoV-2 infection was observed (Fig. 3). One adult patient presented with increased C-reactive protein (CRP) of 30.7 mg/l (norm < 5 mg/l) during COVID-19. However, this patient reported that the clinical course was otherwise mild.

## Discussion

During the SARS-CoV-2 pandemic, centralized patient registries have started to systematically document and report COVID-19 case data. As of 21 May 2021, the European Cystic Fibrosis Society Patient Registry (ECFSPR), which collects COVID-19 cases among CF patients throughout Europe, documented 1158 PCR-confirmed cases in 30 countries [9]. The majority (63%) were adults between 18 and 49 years. In most cases (53%), the course was mild or even asymptomatic. The number of cases with severe or critical course of illness were low (only 36 cases), but 17 patients (1.5%) died. Currently, 6463 patients with cystic fibrosis are living in Germany [10]. As of 18 May 2021, 102 SARS-CoV-2 positive cases have been documented in the German Cystic Fibrosis Registry. These data suggest a low cumulative incidence of 1.6% in the German CF cohort. In comparison, the cumulative incidence in the German population is 4.4% (stand of 18 May 2021; [11]).

In the present study, we report a seroprevalence estimate of 4.2% and, together with the results of molecular testing, a cumulative incidence of SARS-CoV-2 infection of 8.3% among our CF patients. This was surprising at first, since several other studies indicate that COVID-19 incidence estimates appear to be lower in CF than in the general population [12]. Plausible reason for this might be that patients with CF have always paid close attention to infection control. However, the most obvious explanation for the discrepancy between published data and ours could also be the simplest: that frequent testing simply leads to more hits. Another possible reason might be that molecular testing by RT-PCR, though considered as gold standard, is not the leading strategy in large-scale screenings and the performance of rapid tests is far behind [13]. Third, there might be a considerable proportion of silent transmitters, which do not appear in the statistics. The ECFSPR reported 19% asymptomatic courses of SARS-CoV-2 infections in CF patients. In children with CF this quota might even be higher [14]. It is conceivable that a similarly high percentage of SARS-CoV-2 infections remains undetected in the general population.

Limitation of the study might be the retrospective reporting of symptoms, which might have caused recall bias. However, we expect our patients to be alert and sensitive to symptom changes, especially because little was known about the risk COVID-19 poses to patients with CF.

In summary, our observations support preliminary reports that SARS-CoV-2 did not cause worse outcomes in CF lung disease [15–17]. In the meantime, the number of fully vaccinated patients with CF is increasing. Perspectively, continuing to obtain data on antibody responses in patients with CF will be an essential component of determining vaccine efficacy.

## Data Availability

The datasets used and/or analysed during the current study is archived at the JUH and available from the corresponding author on reasonable request.
